# On-demand correction for X-ray response non-uniformity in microstrip detectors by a data-driven approach

**DOI:** 10.1107/S1600577520008929

**Published:** 2020-08-11

**Authors:** Kenichi Kato, Kazuya Shigeta

**Affiliations:** a RIKEN SPring-8 Center, 1-1-1 Kouto, Sayo-cho, Sayo-gun, Hyogo 679-5148, Japan; b JST, PRESTO, 4-1-8 Honcho, Kawaguchi, Saitama 332-0012, Japan; c Nippon Gijutsu Center Co. Ltd, 1-1-1 Kouto, Sayo-cho, Sayo-gun, Hyogo 679-5148, Japan

**Keywords:** flat-field correction, X-ray detectors, X-ray scattering, data science

## Abstract

A statistical approach, which was previously developed to correct scattering data for X-ray response non-uniformity (XRNU) in microstrip detectors, has been improved to significantly reduce the correcting time. The improved algorithm has succeeded in increasing the utilization rate of data acquired for reference intensity to 98%. As a result, the correcting time was reduced from half a day to half an hour, which was shorter than the typical measuring time of a sample. Moreover, the present approach was found to yield better correction results than the previous one. The data-driven approach enabled the on-demand correction for XRNU according to the detector and experimental settings. The present study will encourage the correction of scattering data for XRNU in area detectors.

## Abstract 2

A data-driven approach has been developed to correct scattering data for X-ray response non-uniformity in microstrip detectors on demand. The present study will facilitate the correction in all types of X-ray detectors.

## 1. Introduction   

The dynamic range of an X-ray detector is defined by the difference in X-ray response between detector channels, which was referred to as X-ray response non-uniformity (XRNU) in a previous paper (Kato *et al.*, 2019 ▸). XRNU is one of the systematic errors for individual channels, while it appears to be a random error for different channels. Therefore, it seems difficult to distinguish the intensity variation caused by XRNU (XRNU noise) from that according to the Poisson distribution (Poisson noise). The level of the XRNU noise is fixed by the detector properties, whereas that of the Poisson noise depends on the number of X-ray photons. When the XRNU noise is lower than the Poisson noise, the noise level of the obtained data is in accordance with the photon statistics. In contrast, when the XRNU noise is higher than the Poisson noise, the noise level deviates from photon statistics and is fixed at the level given by the XRNU noise. In other words, the XRNU noise determines the maximum signal-to-noise ratio of the scattering data.

XRNU has been recognized as one of the problems in all types of X-ray detectors such as imaging-plate detectors (Amemiya, 1995 ▸), charge-coupled-device detectors (Williams & Shaddix, 2007 ▸), flat-panel detectors (Skinner *et al.*, 2012 ▸), pixel detectors (Wernecke *et al.*, 2014 ▸), and microstrip detectors (Bergamaschi *et al.*, 2010 ▸). So far, the flat-field approach has been adopted to correct scattering data for XRNU. Basically, the approach needs a uniform reference intensity. A point source and fluorescent emission have been used to illuminate the whole sensitive area of a detector with uniform-intensity X-rays (Hammersley *et al.*, 1995 ▸; Moy *et al.*, 1996 ▸). Based on the difference between the reference and measured intensity, the correction factor of each channel can be obtained. In most cases, the reference intensity deviates from uniformity due to various systematic errors. Nevertheless, the flat-field correction has to be made on the assumption that the reference intensity is perfectly uniform. From our experience, coverage of the conventional approach, which may be considered a hypothesis-driven type, was limited to the case where the level of the XRNU noise was higher than several percent.

An alternative approach, which is based on the statistical estimation of the reference intensity, has been developed to overcome the limitation (Kato *et al.*, 2019 ▸). Such a statistical approach has allowed one to use a non-uniform reference intensity, in which case there is no need for any assumption. It has been reported that the level of the XRNU noise of microstrip detectors was successfully reduced from 1% to 0.1% (Kato *et al.*, 2019 ▸). On the other hand, the approach has a problem with the correcting time. The acquisition of reference data took at least half a day. The long correcting time made it virtually impossible to correct scattering data for XRNU according to the detector and experimental settings, which affect the pattern of XRNU considerably. Accordingly, a significant reduction in the correcting time was required.

Here we report a data-driven approach based on a statistical approach to reduce the correcting time from half a day to half an hour, leading to the on-demand correction for XRNU.

## 2. Methods and applications   

### 2.1. Principle of the statistical approach   

**Figure 1:**
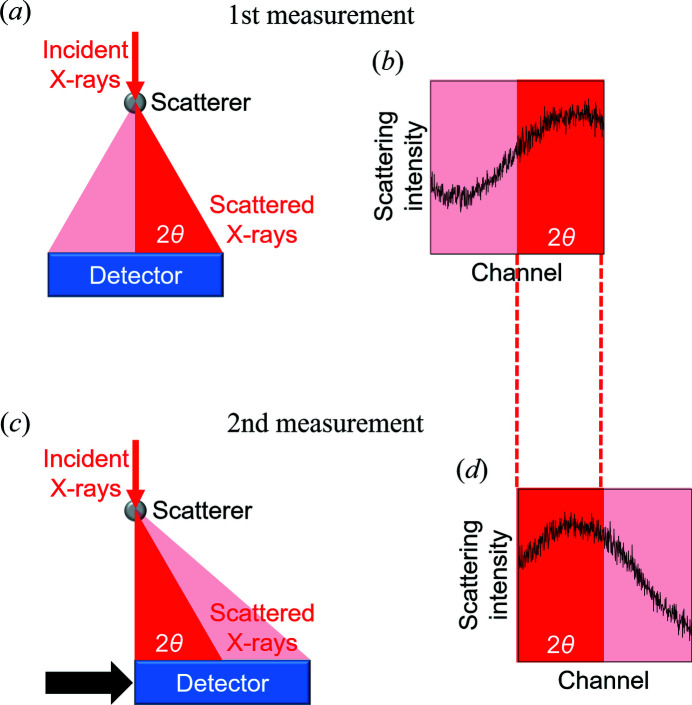
Scheme for the statistical approach to XRNU. Setups for the first (*a*) and second (*c*) measurements. The scattering data acquired by the first (*b*) and second (*d*) measurements.

Let us explain the principle of the statistical approach. Fig. 1 ▸ shows a scheme for acquiring reference data. First, the scattering intensity from a scatterer is measured for a given time by a detector [Fig. 1(*a*) ▸]. Next, the detector is shifted by half of the detector along the arrow, and the second measurement is carried out for the same time as the first measurement [Fig. 1(*c*) ▸]. Figs. 1(*b*) and 1(*d*) ▸ show the scattering data acquired by the first and second measurements, respectively. The first set of data in the 2θ range, which is acquired by the right half of the detector, should be consistent with the second set of data in the same range, which is acquired by the left side of the detector, within the Poisson noise. However, when the XRNU noise is larger than the Poisson noise, the difference between the two sets of data deviates from the level of the Poisson noise. From the difference, the reference intensity in the 2θ range can be statistically estimated. The correction factor for each channel can be found as the ratio of the estimated reference intensity to the observed scattering intensity on each channel.

### 2.2. Three kinds of processes based on the statistical approach   

The previous paper (Kato *et al.*, 2019 ▸) reported two kinds of processes based on the statistical approach: the single-step (SS) and the multi-step (MS) processes, which are characterized by the difference in the step number of estimates of reference intensity. The present paper reports the third kind of process based on the SS process, which is referred to as the optimized single-step (OSS) process hereafter, to make the most use of reference data. Here let us illustrate the three kinds of processes by giving an example assuming that a detector with eight channels is partitioned into eight blocks for simplicity.

#### 2.2.1. SS process   

**Figure 2:**
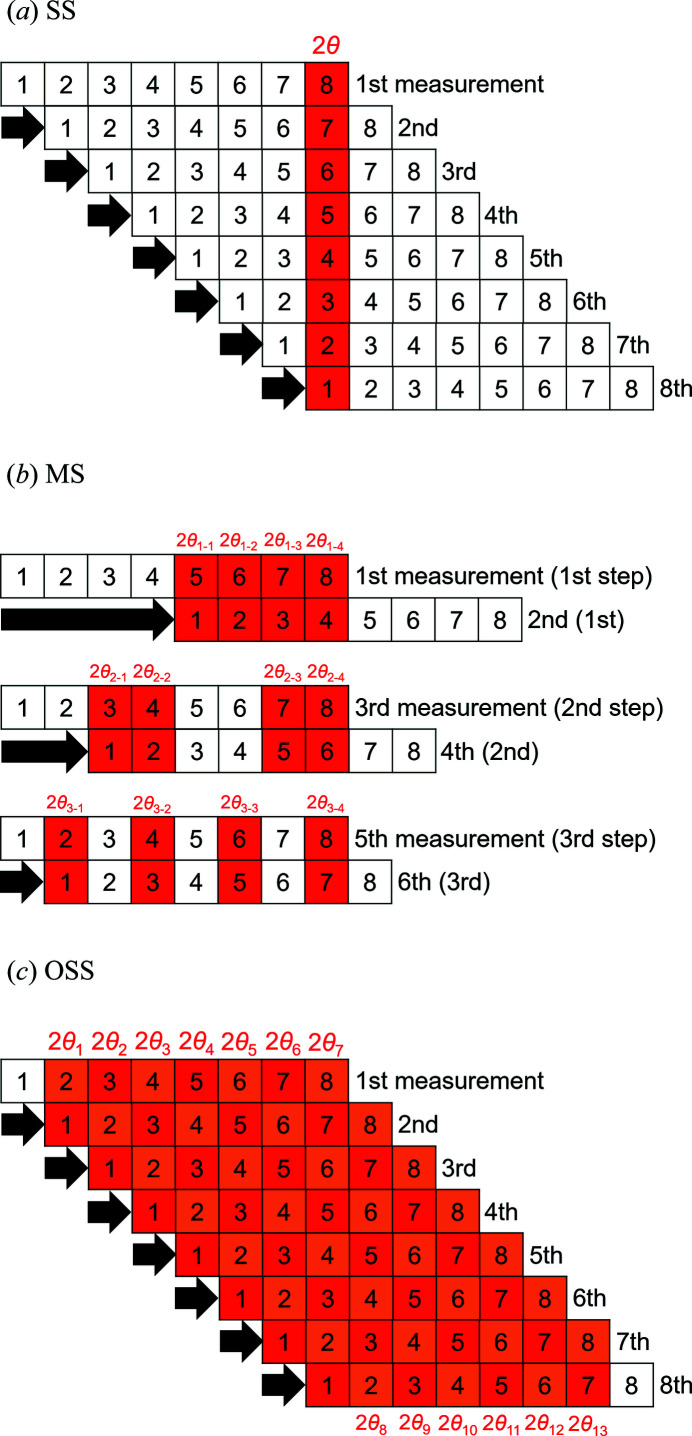
Procedures for acquiring reference data for the SS (*a*), MS (*b*), and OSS (*c*) processes. Each line with eight blocks corresponds to a detector with eight channels. The filled blocks indicate the domain used for estimating reference intensity.

Fig. 2(*a*) ▸ shows a procedure for acquiring reference data for the SS process. First, the scattering intensity from a scatterer is measured for a given time by a detector. After shifting the detector along the arrow by one block, the next measurement is performed for the same time as the first measurement. This procedure is iterated until the eighth measurement. In the SS process, the number of measurements is coincident with the number of blocks. The reference intensity 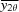 at 2θ can be estimated as the arithmetic mean of eight intensities *y*_2θ_(*i*) measured by eight channels (*i* = 1–8) at 2θ as follows:
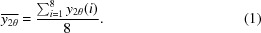
The correction factor *c*_SS_(*i*) for channel *i* can be found as the ratio of the reference intensity 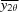 to the measured intensity *y*_2θ_(*i*) as follows:
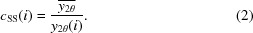
From Fig. 2(*a*) ▸, it is found that the number of blocks used for estimating the reference intensity is 8, whereas those unused is 56.

#### 2.2.2. MS process   

Fig. 2(*b*) ▸ shows a procedure for acquiring reference data for the MS process. First, the scattering intensity from a scatterer is measured for a given time by a detector. After shifting the detector along the arrow by four blocks, which correspond to half of the total length, the second measurement is performed for the same time as the first measurement. A set of the two measurements is iterated until the shift of the detector is one block. Note that the shift of the detector should be set at half of the shift in the prior step [Fig. 2(*b*) ▸]. In the case of the eight-block partitioning, the MS process consists of six measurements and a three-step estimate of the reference intensity.

The reference intensity 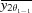, 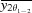, 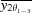, and 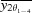 at the first step can be estimated as the arithmetic mean of the two intensities 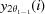, 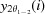, 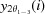, and 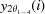 measured by the two different channels (*i* = 1 and 5, 2 and 6, 3 and 7, and 4 and 8) at 2θ_1–1_, 2θ_1–2_, 2θ_1–3_, and 2θ_1–4_, respectively, as follows:
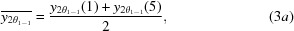


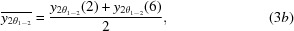


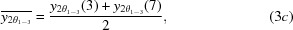


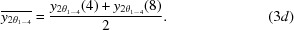
The correction factor *c*_MS1_(*i*) for channel *i* at the first step can be found as the ratio of the reference intensity to the measured intensity as follows:
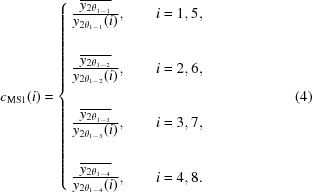



The reference intensity 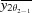, 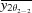, 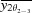, and 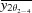 at the second step can be estimated as the arithmetic mean of the two intensities 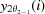, 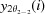, 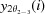, and 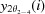 measured by the two different channels (*i* = 1 and 3, 2 and 4, 5 and 7, and 6 and 8) at 2θ_2–1_, 2θ_2–2_, 2θ_2–3_, and 2θ_2–4_, respectively, as follows:
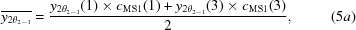


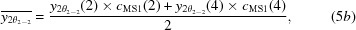


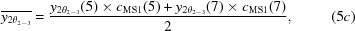


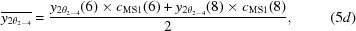
where the key point is that the measured intensity on each channel is corrected by the first-step factor *c*_MS1_(*i*). The correction factor *c*_MS2_(*i*) at the second step can be found as the ratio of the reference intensity to the measured intensity as follows:
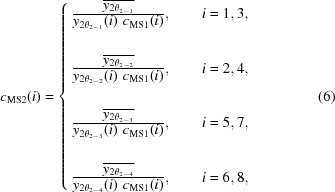
where the key point is that the measured intensity on each channel is corrected by the first-step factor *c*_MS1_(*i*).

The reference intensity 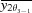, 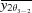, 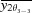, and 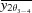 at the third step can be estimated as the arithmetic mean of the two intensities 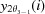, 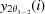, 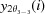, and 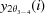 measured by the two different channels (*i* = 1 and 2, 3 and 4, 5 and 6, and 7 and 8) at 2θ_3–1_, 2θ_3–2_, 2θ_3–3_, and 2θ_3–4_, respectively, as follows:
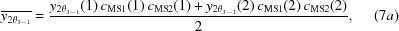


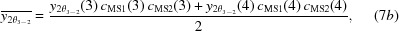


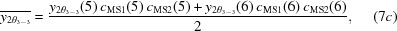


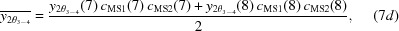
where the key point is that the measured intensity on each channel is corrected by the first-step and second-step factors *c*_MS1_(*i*) and *c*_MS2_(*i*). The correction factors *c*_MS3_(*i*) at the third step can be found as the ratio of the reference intensity to the measured intensity as follows:
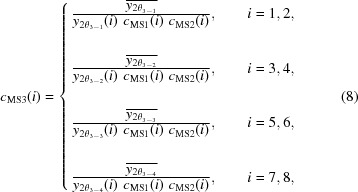
where the key point is that the measured intensity on each channel is corrected by the first-step and second-step factors *c*_MS1_(*i*) and *c*_MS2_(*i*).

The final correction factor *c*_MS_(*i*) for each channel can be obtained by multiplying the three preliminary factors *c*_MS1_(*i*), *c*_MS2_(*i*), and *c*_MS3_(*i*) at each step as follows:
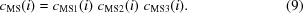
From Fig. 2(*b*) ▸, it is found that the number of blocks used for estimating the reference intensity is 24, whereas those unused is 24.

#### 2.2.3. OSS process   

Fig. 2(*c*) ▸ shows a procedure for acquiring reference data for the OSS process, which is the same with the SS process [Fig. 2(*a*) ▸]. The SS process has a single point of reference intensity, whereas the OSS process has 13 points of reference intensity. The ‘local’ correction factor 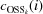 for channel *i* can be estimated based on the reference intensity at 2θ_*k*_ in the same way as the SS process as follows:
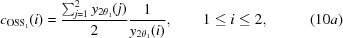


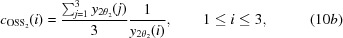


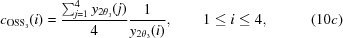


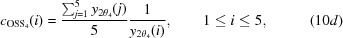


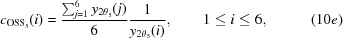


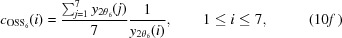


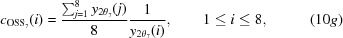


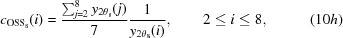


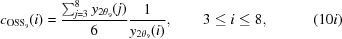


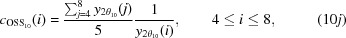


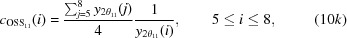


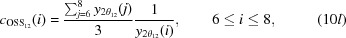


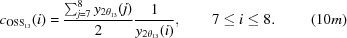
The ‘global’ correction factor *c*_OSS_(*i*) for channel *i* can be estimated as the weighted mean of the multiple ‘local’ correction factors 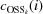 as follows:
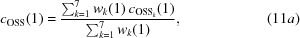


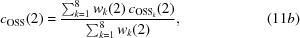


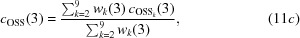


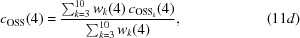


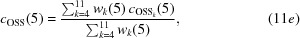


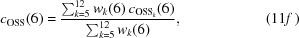


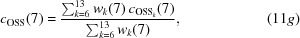


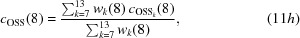
where *w_k_*(*i*) is the weight for 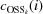 expressed by 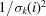, where *σ_k_*(*i*) is the sample standard deviation of 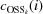. From Fig. 2(*c*) ▸, it is found that the number of blocks used for estimating reference intensity is 62, whereas those unused is 2.

### 2.3. Applications of the three kinds of processes to OHGI   

The three kinds of processes were applied to data acquired by the total-scattering measurement system (Kato *et al.*, 2019 ▸) installed at the RIKEN Materials Science beamline BL44B2 (Kato *et al.*, 2010 ▸; Kato & Tanaka, 2016 ▸) of SPring-8, which we named ‘OHGI’ (Overlapped High-Grade Intelligencer). OHGI is composed of 15 microstrip modules [MYTHEN (Schmitt *et al.*, 2003 ▸), DECTRIS Ltd], each of which has 1280 channels. The details of the system were described in the previous paper (Kato *et al.*, 2019 ▸). The examples in Section 2.2 show the model case where a block contains a single channel. In the real case where a block contains multiple channels, the correction factor within a block can be calculated for each channel using the reference intensity at each 2θ. In the present study, each module was partitioned into 128 blocks, each of which has ten channels. The number of measurements in the SS and OSS processes was 128, whereas that in the MS process was 14, which corresponds to a seven-step estimation. A SiO_2_ glass rod with a diameter of 3.5 mm was used as a scatterer for reference data since it can give high scattering intensity in a wide range of 2θ. As a result, reference data for the 15 modules were able to be acquired simultaneously, which means that the correcting time does not depend on the number of modules. The wavelength of incident X-rays was set at 0.45 Å. The energy threshold of OHGI was set at 13.8 keV, which is half of the energy of incident X-rays. Correction factors for the SS, MS, and OSS processes were obtained by extending equations (1)–(2), (3)–(9), and (10)–(11) to the case of 128 blocks, respectively. The 16 channels from both ends of each module were excluded from the calculation of the correction factors because of the unfavourable intensity for the SS and MS processes, whereas 128 channels for the OSS process.

## 3. Results   

### 3.1. Comparison of the results corrected by the three kinds of processes   

**Figure 3:**
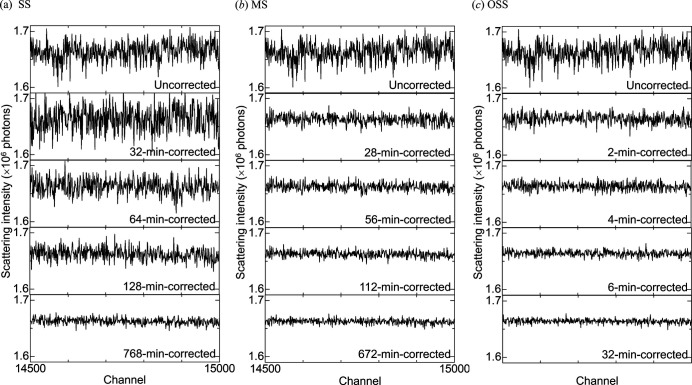
Correcting-time evolutions of the SiO_2_ scattering data corrected by the SS (*a*), MS (*b*), and OSS (*c*) processes. Channels from 14500 to 15000 were selected from 19200 channels for comparison. The top panels display the identical uncorrected data.

To compare the results corrected by the three kinds of processes, the reference data for each process were acquired in different times, which were fixed based on multiples of the number of measurements in each process. The correction factors obtained from the reference data were applied to the scattering data of a SiO_2_ glass rod with a diameter of 3.5 mm, which was measured for an hour to give an intensity of the order of 10^6^ photons independent of the reference data. Fig. 3 ▸ shows the correcting-time evolution of the scattering data of SiO_2_ for each process. In the SS process [Fig. 3(*a*) ▸], the 32 min correction increased variations in the uncorrected data, whereas the longer-time corrections decreased variations. In the MS process [Fig. 3(*b*) ▸], the 28 min correction rapidly decreased variations, while the longer-time corrections gradually decreased variations with correcting time. Figs. 3(*a*) and 3(*b*) ▸ indicate that the 112 min correction in the MS process was comparable with the 768 min correction in the SS process. In the OSS process [Fig. 3(*c*) ▸], only the 2 min correction rapidly decreased variations. Figs. 3(*b*) and 3(*c*) ▸ indicate that the 32 min correction in the OSS process was comparable with the 672 min correction in the MS process.

**Figure 4:**
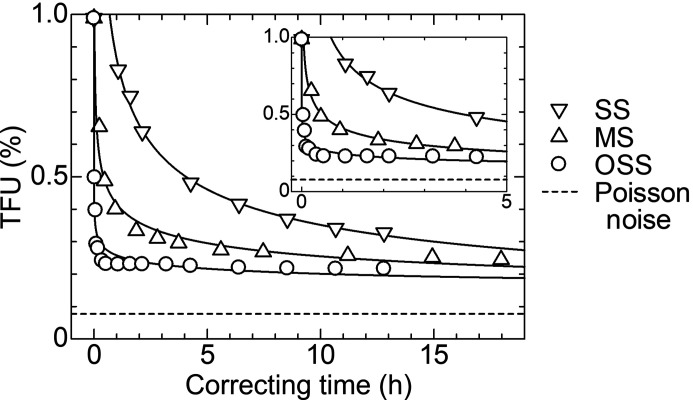
Correcting-time evolutions of the TFU evaluated from the SiO_2_ scattering data based on the SS, MS, and OSS processes. The inset shows the correcting time from 0 h to 5 h. Channels from 14500 to 15000, which is shown in Fig. 3 ▸, were selected for evaluation. The ideal TFU, which is based on the Poisson noise, is shown for reference. The TFU at 0 h corresponds to that of the uncorrected data.

To examine the results in Fig. 3 ▸ in detail, the correcting-time evolution of the SiO_2_ scattering data was evaluated in terms of an index of the noise level, *i.e.* the total fractional uncertainty (TFU), which was introduced in the previous paper (Kato *et al.*, 2019 ▸) and was given as
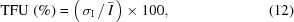
where *Ī* and σ_I_ are the arithmetic mean and the sample standard deviation of the scattering intensities measured by selected channels, respectively. Fig. 4 ▸ shows the correcting-time evolution of the TFU for the three kinds of processes. The TFU of the SS process did not reach a plateau, while that of the MS and OSS processes reached a plateau in half a day and half an hour, respectively. The SS and MS processes reached a TFU of 0.33% in 768 min and 112 min, respectively. Meanwhile, the OSS process reached a TFU of 0.29% in 6 min. None of the processes reached the level of the Poisson noise. The time for reaching a plateau indicates that the correction efficiency in the OSS process was 24 times as high as that in the MS process. In addition, the OSS process was found to yield better results than the MS process.

### 3.2. ‘ReLiEf’ effects of the OSS process   

**Figure 5:**
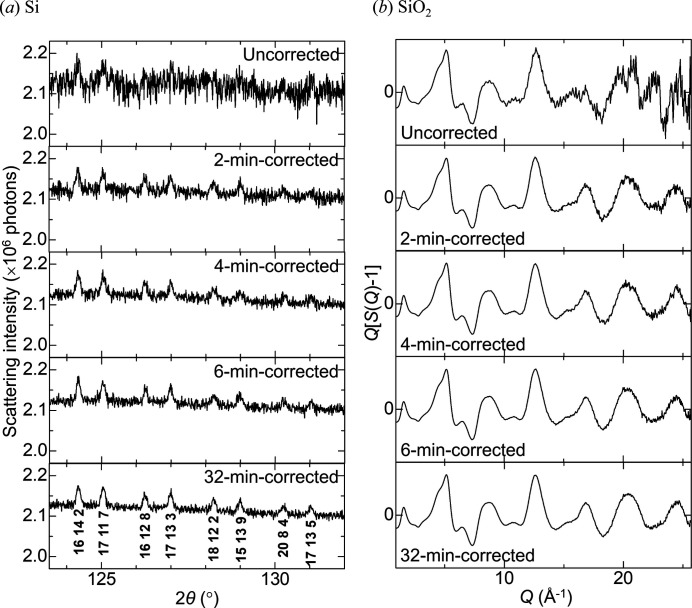
Correcting-time evolutions of the observed scattering intensity of Si (*a*) and *Q*[*S*(*Q*) − 1] of SiO_2_ (*b*) based on the OSS process. The axes of abscissa in (*a*) and (*b*) are 2θ and *Q*, respectively. The top panels display the uncorrected data. At the bottom of (*a*), the observed peaks are indicated by the primary index.

To examine the correction effect of the OSS process on Bragg and diffuse scattering, the scattering data of Si (NIST Standard Reference Material 640d) and SiO_2_ were corrected in different correcting times, respectively. Si powders in a glass capillary of 0.3 mm in diameter and a SiO_2_ glass rod of 3.5 mm in diameter, which was identical to the reference scatterer for correction, were measured for 862 min and 60 min, respectively, to give an intensity of the order of 10^6^ photons at higher angles. The corrected and uncorrected scattering data of SiO_2_ were integrated by ±10 channels and converted into *Q*[*S*(*Q*) − 1] (Yang *et al.*, 2014 ▸), where *Q* is the magnitude of the scattering vector and *S*(*Q*) is the structure factor. Figs. 5(*a*) and 5(*b*) ▸ show the correcting-time evolution of the observed scattering intensity of Si and *Q*[*S*(*Q*) − 1] of SiO_2_. No Bragg peaks were observed in the uncorrected data of Si [Fig. 5(*a*) ▸]. However, some peaks were visible in the 2 min-correction data. These peaks became clear with correcting time. The 32 min-correction data indicates that two peaks, 20 8 4 and 17 13 5, which are four orders of magnitude lower than the highest-intensity peak 1 1 1, were clearly observed. The noise of the uncorrected data of SiO_2_ increased with *Q* [Fig. 5(*b*) ▸]. The noisy part improved significantly with correcting time. The 32 min-correction data indicates that the diffuse scattering at high *Q* was clearly observed. These results inspired us to term the statistical approach ‘ReLiEf’ (Response-to-Light Effector), because the signal stood out in ‘relief’ against the noise.

### 3.3. Dynamic range of OHGI   

**Figure 6:**
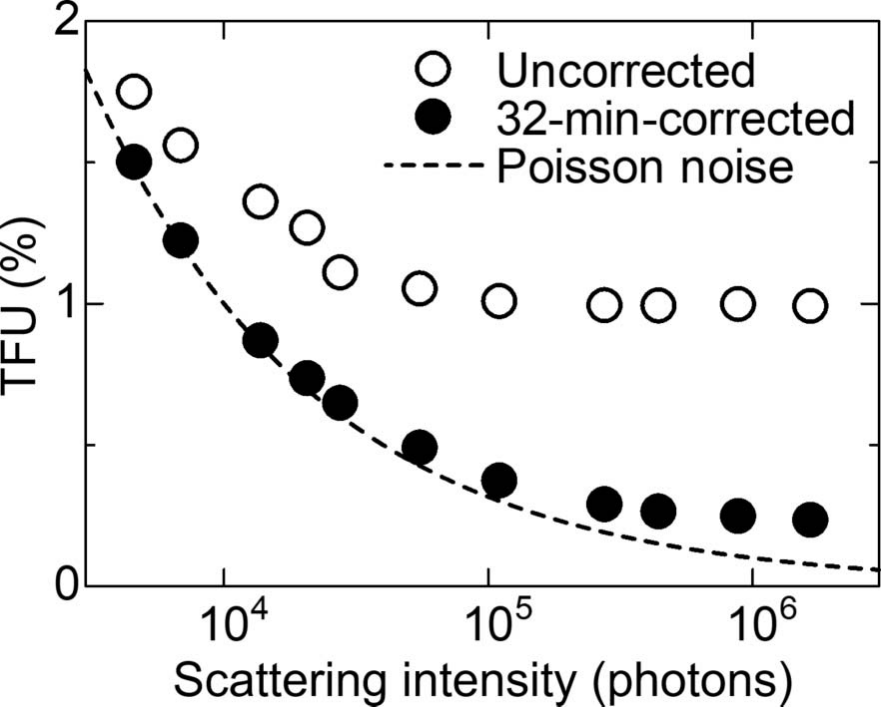
TFUs of the uncorrected and corrected scattering data of SiO_2_ as a function of observed scattering intensity. The corrected data is based on the 32 min correction by the OSS process. Channels from 14500 to 15000, which is shown in Fig. 3 ▸, were selected for evaluation. The TFU curve based on the Poisson noise is shown for comparison.

Fig. 6 ▸ shows the TFUs of the uncorrected and corrected scattering data of SiO_2_ as a function of observed scattering intensity. The TFU of the uncorrected data increasingly deviated from that of the Poisson noise with scattering intensity and reached a plateau of 1%, which corresponds to that of the Poisson noise at 10^4^ photons. The results indicate that the dynamic range of OHGI was limited to 10^4^ without correction. In contrast, the TFU of the corrected data was close to that of the Poisson noise over a wide range of scattering intensity. The lowest value of TFU indicates a dynamic range over 10^5^. These findings demonstrate that the OSS process improved the dynamic range by one order of magnitude in half an hour.

## 4. Discussion   

As shown in Fig. 4 ▸, the TFU by the MS and OSS processes reached a plateau in half a day and half an hour, respectively. The difference in correcting time shows that the correction efficiency improved by a factor of 24. A large part of the improvement may be explained from the number of blocks used for correction. In the present study, each module of OHGI was partitioned into 128 blocks for correction. According to Fig. 2(*b*) ▸ and Section 2.2.2, the utilization rate of reference data in the MS process is found to be 50% independent of the number of blocks. In the case of the 128-block partitioning, 896 blocks (= 1792 blocks × 0.5) out of the total number of blocks (1792 = 128 blocks × 14 measurements) were utilized for the MS process. Meanwhile, according to Fig. 2(*c*) ▸ and Section 2.2.3, the OSS process utilized 16128 blocks (= 16384 blocks − 256 blocks) out of the total number of blocks (16384 = 128 blocks × 128 measurements), where the subtraction means that either block of the module was excluded from calculations. The utilization rate in the OSS process reached 98%, whereas that in the SS process reached only 1%. That is why the OSS process is described as a data-driven approach in the Title and the Abstract of this paper. The number of blocks for the OSS process was 18 times as large as that for the MS process. Accordingly, a large part of the improvement is found to be due to the difference in the number of blocks for correction.

The remaining part of the improvement might be understood as follows. The statistical approach is based on the condition that temporal fluctuations in intensity from a reference scatterer are negligible. In the previous paper (Kato *et al.*, 2019 ▸), the divided-accumulation technique was introduced to reduce the effect of temporal fluctuations in the SS and MS processes. However, the OSS process did not need such a technique since fluctuations were negligible for half an hour. It should be emphasized that the data-driven approach by the OSS process plays an important role in meeting the requirement for the statistical approach.

## 5. Conclusion   

Making the best use of reference data has succeeded in reducing the correcting time from half a day to half an hour, which was shorter than the typical measuring time of a sample. Consequently, the data-driven approach based on the OSS process has allowed one to correct the XRNU in microstrip detectors on demand according to the detector and experimental settings. Finally, let us mention the application of the data-driven approach to area detectors such as pixel detectors and flat-panel detectors. In contrast to the flat-field approach, the statistical approach is characterized by the fact that the correcting time is not affected by the dimensions of a detector system since a reference object scatters X-rays in all directions. The present study will stimulate one to correct data measured by all types of detectors to make full use of the dynamic range.
